# Frequency-Dependent Effects on Coordination and Prefrontal Hemodynamics During Finger Force Production Tasks

**DOI:** 10.3389/fnhum.2021.721679

**Published:** 2021-10-18

**Authors:** Dayuan Xu, Narae Shin, Sungjun Lee, Jaebum Park

**Affiliations:** ^1^Department of Physical Education, Seoul National University, Seoul, South Korea; ^2^Institute of Sport Science, Seoul National University, Seoul, South Korea; ^3^Advanced Institute of Convergence Technology, Seoul National University, Suwon, South Korea

**Keywords:** multi-finger synergy, uncontrolled manifold hypothesis, prefrontal cortex, near-infrared spectroscopy, functional connectivity, frequency of cyclic action

## Abstract

Behavioral stability partially depends on the variability of net outcomes by means of the co-varied adjustment of individual elements such as multi-finger forces. The properties of cyclic actions affect stability and variability of the performance as well as the activation of the prefrontal cortex that is an origin of subcortical structure for the coordinative actions. Little research has been done on the issue of the relationship between stability and neuronal response. The purpose of the study was to investigate the changes in the neural response, particularly at the prefrontal cortex, to the frequencies of isometric cyclic finger force production. The main experimental task was to produce finger forces while matching the produced force to sine-wave templates as accurately as possible. Also, the hemodynamics responses of the prefrontal cortex, including oxy-hemoglobin concentration (ΔHbO) and the functional connectivity, were measured using functional near-infrared spectroscopy. The frequency conditions comprised 0.1, 1, and 2 Hz. The uncontrolled manifold (UCM) approach was applied to compute synergy indices in time-series. The relative phase (RP), the coefficient of variation (CV) of the peak and trough force values were computed as the indices of performance accuracy. The statistical parametric mapping (SPM) was implemented to compare the synergy indices of three frequency conditions in time-series. A less accurate performance in the high-frequency condition was caused not by the RP, but mainly by the inconsistent peak force values (CV; *p* < 0.01, $\eta_{\text{p}}^{2}$ = 0.90). The SPM analysis revealed that the synergy indices were larger in the low-frequency than in high-frequency conditions. Further, the ΔHbO remained unchanged under all frequency conditions, while the functional connectivity decreased with an increase in the frequency of cyclic force production. The current results suggested that the concurrent activation of the prefrontal region mainly depends on the frequency of cyclic force production, which was associated with the strength of stability indices and performance errors. The current study is the first work to uncover the effect of frequency on the multi-finger synergies as to the hemodynamic response in the prefrontal cortex, which possibly provides a clue of the neural mechanism of synergy formation and its changes.

## Introduction

Exploring behavioral stability is a common performance goal of many daily life activities such as holding and moving a glass of water while preventing spilling over, as well as experimental tasks performed in a laboratory. Notably, stable performance partially refers to the reproducible mechanical outcomes of important performance variables across multiple attempts (Newell and Carlton, 1988; McIntyre et al., 1996; Hasan, 2005). In this sense, lowering net outcome variability across repetitive attempts is a prerequisite for achieving high performance stability (Scholz and Kelso, 1989; Kipp and Palmieri-Smith, 2012; Rajachandrakumar et al., 2018). As evidenced by a series of experiments based on the principle of motor abundance (Jaric and Latash, 1999; Latash et al., 2002a; Shim et al., 2003; Latash, 2012), low net outcome variability could be the consequence of the combined effect of the relatively high variability of individual elements. Specifically, this variability comprises a host of solutions from an abundant set of elements, where a significant fraction of variability (i.e., variance) is channeled into the subspace corresponding to the required mechanical actions (Reschechtko et al., 2014; Ambike et al., 2016). The uncontrolled manifold (UCM) framework has been widely used to quantify the stability indices of various mechanical outcomes, along with the trial-to-trial variability of the involved elements (see reviews in Scholz and Schöner, 1999; Latash, 2008a). Stability in human behavior refers, in part, to the central and peripheral ability to stabilize salient performance variables by a proper organization of multiple elements involved in a specific behavior, which is well in accordance with the classical definition of stability (Taga, 1995; Patla, 2003). The computational process of the UCM analysis includes the compartmentalization of two lower-dimensional subspaces in the ***n***-dimensional space of elemental variables. The first subspace, so-called UCM space, is concerned with the manifold, where changes in the elements’ actions have no net mechanical effect, and the second subspace is the orthogonal (ORT) space to the UCM subspace, where the elements’ actions have a net mechanical effect. Note that the computation of variance within the two subspaces was performed in linearized subspaces (i.e., a linear approximation, Latash, 2008a). If most of the force variance of individual effectors is confined within the UCM (i.e., V_UCM_) and if the variance observed in the ORT space (i.e., V_ORT_) is relatively small, we may conclude that the net force is stabilized by the co-varied adjustment of individual forces by the effectors. This phenomenon was further confirmed by studies using external perturbation (Scholz et al., 2007; Krishnan et al., 2011, 2012), where the understanding of experimental observation is accompanied by the definition of stability in classical mechanics (Taga, 1995; Hasan, 2005; Bruijn et al., 2013). Moreover, stable performance, to some extent, is the basic premise of accurate and precise performance; however, there are diverse approaches and standards for the quantification of stability and accuracy (Hasan, 2005; Winter, 2009; Kim et al., 2018).

Recent studies on the coordination of multi-finger forces showed that performance errors with respect to the constrained target values and performance reproducibility were highly correlated with the stability indices of the net outcome variable by means of co-varied adjustment of an abundant set of elements (Park et al., 2010; Park et al., 2012a; Park and Xu, 2017; Kim et al., 2018). Such co-varied organization of elemental variables has been termed “*synergy*” (Latash, 2008a). Notably, the co-variation patterns (i.e., across-trial variability) differ depending on a particular task or associated constraints; thus, synergy, as a stability measure, possibly represents the purposeful or coordinated neural activities necessary for stabilizing the constrained or hypothetical performance variables (Latash, 2008b, 2016; Bruton and O’Dwyer, 2018). One of the main properties of a cyclic action is its frequency, which reflects the rate of change in the outcome variables. The frequency of cyclic actions has been claimed to affect both performance stability and variability (Yoshinaga et al., 2000; Stegemoller et al., 2009; Roemmich et al., 2012) as well as the changes in the neural involvement, especially on the basal ganglia and cerebellum (Ivry and Keele, 1989; Spencer et al., 2003).

A significant number of previous studies have addressed the relationship between the frequency of human actions and stability, either across repetitive trials or over time-series (Danion et al., 2003; Zhang et al., 2007; Friedman et al., 2009; Bailey et al., 2018; Floría et al., 2019). The results commonly indicate that relatively high-frequency actions induce large variability, mainly associated with performance errors during repetitive assembly tasks (Bosch et al., 2011). Furthermore, a mathematical model verified that movement frequency played a crucial role in the coordination among finger actions and the overall performance stability (Friedman et al., 2009). The generation of voluntary actions is assumed to be completed by a hierarchical control scheme (Schöner et al., 1995), and the frequency component is considered to be associated with a high-level control. Thus, it is highly probable that changes in the frequency of cyclic actions require the appropriate adjustment of neuronal activity that causes voluntary actions. However, the neural origin of the aforementioned observations regarding the frequency-dependent features of motor outcomes remains unknown.

Studies on the effect of the frequency of various motor behaviors on movement-related cortical activation have mainly focused on motor tasks including two limbs, two fingers, or two joint actions (Kuboyama et al., 2005; Wang et al., 2007, 2017) and walking with the help of various brain imaging techniques (Harada et al., 2009; Kim et al., 2016, 2017; Nordin et al., 2019). Notably, the responses of neural structures within the corticobasal-thalamo-cortical circuit are commonly increased with an increase in the movement frequency (Jenkins et al., 1997; Turner et al., 1998; Toma et al., 2002; Agnew et al., 2004; Suzuki et al., 2004); however, the oscillatory cortical responses are desynchronized during relatively fast movement (Bulea et al., 2015). The corticobasal-thalamo-cortical circuit, which originates from the prefrontal cortex and the primary motor cortex, is one of the main contributors to the coordination of voluntary movement. In other words, the prefrontal region is anatomically defined to have reciprocal connections with a wide spectrum of brain structures, including the basal ganglia, premotor cortex, and supplementary motor area (Fuster, 2015; Miller and Cummings, 2017). The essential functions of the prefrontal region include the coordinated execution of new forms of organized goal-directed action (Killcross and Coutureau, 2003; Hasselmo, 2005; Hart et al., 2018). Recently, functional near-infrared spectroscopy (fNIRS) has been used to quantify brain activity associated with hemodynamic responses (Bunce et al., 2006; Ferrari and Quaresima, 2012; Scholkmann et al., 2014; Yeung and Chan, 2021). This implies that neuronal activity is associated with localized cerebral blood flow, which has been termed neurovascular coupling (Chiarelli et al., 2017; Kaplan et al., 2020). In particular, fNIRS quantifies the oxygen-saturated hemoglobin (i.e., oxy-hemoglobin, O_2_Hb) and deoxygenated hemoglobin (i.e., deoxyhemoglobin, HHb) in the blood circulating to the prefrontal region. In addition, the observation of concurrent activations of different locations within the prefrontal region, the so-called functional connectivity (Murrough et al., 2016; Yu et al., 2020), may provide information about the interconnected neural activation for voluntary movement in the prefrontal region and its changes with the frequency changes of cyclic actions. Therefore, it is possible that the prefrontal circuitry is modulated by the frequency of cyclic actions. However, little is known about the relationship between the prefrontal response and the organization of multi-element actions in humans such as the patterns of co-variation among a set of elements. The role of the prefrontal structure and its changes during voluntary actions is rarely investigated; thus, the measure of the prefrontal area may provide a meaningful clue about the neural origin of the synergy formation and its effect on the frequency of cyclic actions in humans.

The current study attempts to examine the effect of cyclic action frequency on the synergy of multi-finger actions and the hemodynamic responses in the prefrontal region during accurate cyclic-force-production tasks in an isometric condition (i.e., static condition). On the basis of prior knowledge and experimental outcomes, we hypothesized that: (1) performance accuracy decreases with an increase in frequency (i.e., less accurate); (2) the synergy indices show frequency dependence, wherein a larger error variance occurs under higher frequencies; and (3) the indices of prefrontal hemodynamics, including the changes in oxy-hemoglobin (O_2_Hb) concentration, ΔHbO, and functional connectivity, increase with the frequency of cyclic finger forces.

## Materials and Methods

### Participants

The advertisement of the study participants was posted on a web-board of Seoul National University. The inclusion criteria comprised no medical history of neurological disorders, injuries of upper extremities, and vision. In particular, the experimental tasks required the proper acquisition of visual information on the computer screen, all volunteers who passed the Freiburg Visual Acuity and Contrast Test (FrACT; Bach, 1996) were selected for participants (i.e., above 1.0 decimal scale as the normal condition of vision by 20/20 visual acuity scale). A power analysis using G*Power (Faul et al., 2007) was performed to estimate a prior sample size, which suggested recruiting at least nine participants in order to reach an effect size (d) greater than 0.7 with at least 80% power and *α* = 0.05 as type-I error rate to detect significant differences between the conditions. Twelve volunteers were recruited, and three of them did not satisfy the inclusion criteria. Thus, nine right-hand dominant young males (age, 30.3 ± 2.7 years; height, 167.49 ± 6.53 cm; weight, 69.39 ± 15.73 kg) participated in the experiment. The Seoul National University Institutional Review Board (IRB) approved the use of customized experimental protocols and compatible devices related to the current behavioral tasks (e.g., force transducers and fNIRS devices). After providing information about the study, we requested all the participants to sign a consent form approved by the IRB at Seoul National University (IRB No. 2007/002-028). The original signed consent form was retained in the experimental records, and a copy of the signed consent form was provided to the participants.

### Apparatus

**Figure 1:**
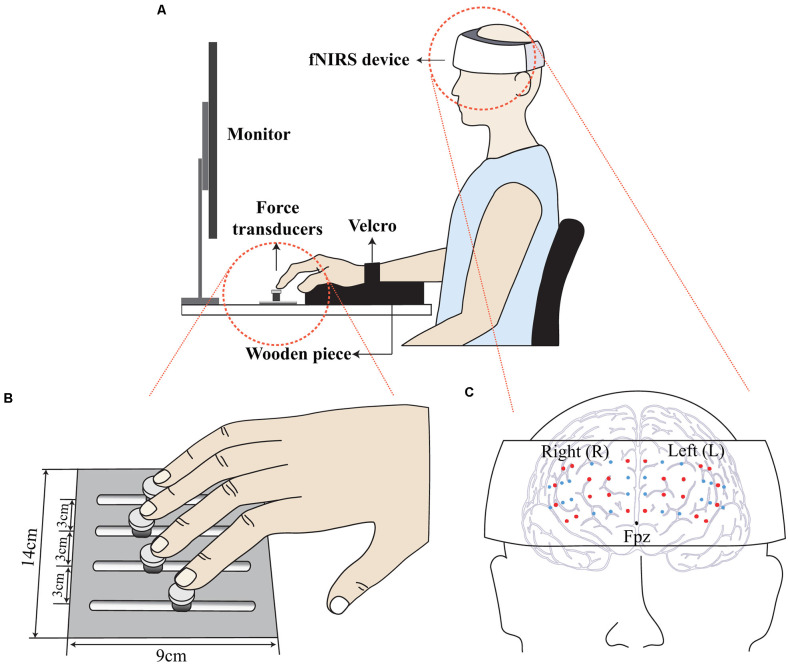
Illustration of the experimental setup. Participants wore a wearable fNIRS device, and the computer screen visualized the real-time force and the force template. The participant’s wrist was held stationary with Velcro straps while a wooden cylinder supported the palm **(A)**. Four force transducers were attached to a frame **(B)**. A wearable fNIRS device located just above the nasion (Fpz), and the probes (small dots) were firmly contacted on the prefrontal region **(C)**.

Four force transducers (Nano-17, ATI Industrial Automation, Garner, NC) were attached to a customized experimental frame (140 × 90 × 5 mm^3^) to independently measure the pressing forces (i.e., *z*-axis forces) produced by each of four fingers (Figure 1B). The transducers’ surfaces were covered with sandpaper to provide sufficient friction to the fingertips. There were four slots in an anterior-posterior direction on the panel to adjust the transducer position to the hand and finger size of individual participants. The mediolateral distance between slots was fixed at 3.0 cm (Figure 1B). The 3.0 cm value was a typical distance reported in the previous hand/finger pressing experiments, which possibly ensured a comfortable hand posture during pressing with fingers (Park and Xu, 2017; Kim et al., 2018; Kong et al., 2019). The experimental frame attached to the transducers was mechanically fixed on an immovable table. Four analog signals from the transducers were digitized with 16-bit analog-digital converter (USB-6225, National Instrument, Austin, TX, USA) *via* a customized LabVIEW program (LabVIEW 8.0, National Instrument, Austin, TX, USA). The sampling rate of force signals was set to 200 Hz.

Depth-dependent hemodynamic responses in the prefrontal cortex were recorded using a wearable fNIRS device (NIRSIT, OBELAB, Seoul, Korea) at a sampling rate of 8.138 Hz. The fNIRS probes were in contact with the forehead (Figure 1C). The effect of ambient light was minimized, as confirmed by monitoring the channel quality (refer to the data processing section for more details) throughout the experiment. The length of the source-detector (SD) was 30 mm as in previous reports (Scholkmann et al., 2014), and a total of 48-channel signals covering the majority of the prefrontal cortex were measured.

### Experimental Procedure

All experimental procedures were performed in accordance with the relevant guidelines and regulations of the IRB. Participants sat in a height-adjustable chair with a computer screen placed at eye level. The right upper arm was positioned in the wrist-forearm brace and strapped with Velcro to prevent excessive forearm and wrist movement during the production of pressing finger forces. A wooden piece was placed underneath the participant’s palm to ensure a fixed finger configuration during the experiment (Figure 1A). Before each trial, the subjects were asked to put their fingertips on the center of the corresponding transducers. Prior to each trial, all transducer signals were set to zero at a relaxed moment; thus, the gravitational effect was excluded, resulting in only active pressing finger forces being measured during data acquisition.

The experiment consisted of auxiliary finger force production tasks as well as the main task as a cyclic–force-production task in an isometric condition. The auxiliary tasks included maximal voluntary contraction (MVC) tasks, which involved single-finger pressing tasks and a multi-finger pressing task, and single-finger ramp-force-production tasks for individual fingers. The MVC pressing forces of the individual fingers or all four fingers (MVC_IMRL_) were measured for each participant, and these values were further used to normalize the individualized target force values for the main task. The ramp-force-production tasks were designed to configure an interdependency matrix (i.e., enslaving matrix, **E**) by measuring unintentional force production by non-task fingers, according to the nearly linear relationship between changes in individual finger force and the total force (Latash et al., 2002a). In the ramp-force-production task, participants were asked to produce a force ramp pattern *via* one finger force from 5% to 25% of the finger’s MVC for 6 s after maintaining 5% of that MVC for 2 s. Participants were instructed to focus on the force produced by the task finger and on the template displayed on the computer screen and to ignore the force produced by non-task fingers while keeping all non-task fingers on the corresponding sensors.

The main task of the current study was cyclic-force-production using all four fingers. Participants were asked to produce a smooth sine-wave total finger force (F_TOT_) while wearing the fNIRS device (Figure 1A). Note that the inherent time delay of the hemodynamics response was about 10 s (Fazli et al., 2012), the data acquisition of the fNIRS measure for each trial lasted until 10 s after the force measurement ended. The participants were asked to follow the real-time F_TOT_ on the template displayed on the computer screen while minimizing any unnecessary behaviors such as head motion and speaking that possibly affected the fNIRS measure. The fraction of the time window display on the screen was 10 s, from −5 s to + 5 s with respect to 0 s, that represented the real-time moment of actual force production. The force templates consisted of a 10 s steady force line with a 15% MVC_IMRL_ value, followed by an 80 s sinusoidal force template with three frequency conditions, including 0.1 Hz, 1 Hz, and 2 Hz and the same amplitude (i.e., from 10% to 20% of the MVC_IMRL_ value). The 10 s steady-state phase was designed to elicit a stable baseline hemodynamic state before cyclic force production (Bajaj et al., 2014). Each participant was allowed to practice for about 30 min, which possibly minimized the learning effect during the experiment. After the orientation session and sufficient rest, each participant performed three trials; they were allowed to rest for 5 min after the completion of a single trial. The three frequency conditions were block-randomized across all the participants.

### Data Processing

#### Finger Force Data Analysis

Customized analysis codes (MATLAB, MathWorks, Natick, MA, USA) were written for the finger force data analyses during both the auxiliary and main tasks. Prior to the variable computation, individual finger force data were low-pass filtered using a 4th-order Butterworth filter with a 10 Hz cut-off (Park and Xu, 2017). The force data collected during the main task, that is, the cyclic force production task, were divided into half-cycles of force increase (F_UP_) and force decrease (F_DW_; Park et al., 2012b). The initiation and termination of the F_UP_ and F_DW_ phases were identified as the time when the absolute value of the rate of force change (|dF/dt|) dropped <5% of the peak value of |dF/dt| before and after the time of the peak value of |dF/dt|. Furthermore, the peak and trough values of the F_TOT_ were detected for each cycle. For both F_UP_ and F_DW_ data, the following two criteria were used to identify the erroneous cycles, which were excluded from further analysis: (1) the difference in force magnitude between the produced positive (peak) and negative peak (trough) values of F_TOT_ to the prescribed values of the force template >±5%, and (2) the deviation of cycle duration of the produced force profile from the template >15%. The accepted force data for a single cycle were resampled to 100 data points for both F_UP_ and F_DW_ phases using cubic spline interpolation. As with the indices of performance accuracy, first, the coefficients of variation (CVs) of the peak (CV_peak_) and trough (CV_trough_) values with respect to the prescribed peak or trough values provided by the cyclic force template across the accepted cycles were computed. Thus, the CVs (i.e., standard deviation/arithmetic mean) could be positive or negative depending on the relative values of the actual forces to the prescribed force. Second, the relative phases (RPs) for each F_UP_ and F_DW_ phase were estimated as the index of phase synchronization between the time-series of the total force (F_TOT_) and the prescribed template force. The instantaneous phases of these two signals were calculated using the Hilbert transform (Pikovsky and Rosenblum, 2003). Briefly, the RP is the measure of phase synchronization between two signals (Rybski et al., 2003); therefore, the RP values ranged from 0° (i.e., in-phase synchronization) to 180° (i.e., anti-phase synchronization). Absolute RP values were used because the focus of the RP computation, in this study, was not time lead-lag relations. In addition, the RP values were averaged across samples during the F_DW_ and F_UP_ phases, respectively, for each subject and frequency condition.

The UCM approach was used to compute an index of multi-finger force-stabilizing synergy (Scholz and Schöner, 1999; Latash et al., 2001; Park et al., 2012a). Sets of time-aligned force data for each subject and condition, which are assumed to be hypothetical commands to fingers for force production, were converted to the mode vector (**m**). Briefly, a mode vector reflects the intended finger involvement of all four fingers by commands, and computed by multiplying two matrices, the inverse of the enslaving matrix (**E**) and the individual finger force vector (Equation 1). The interdependency matrix was computed from the single-finger ramp-force-production task for each individual finger (see Park and Xu, 2017 for more computational details).


(1) $$\text{m}={\left[E\right]}^{-1}F;F={\left[f_{I},f_{M},f_{R},f_{L}\right]}^{T}$$

The UCM represents the combinations of individual finger forces that do not alter F_TOT_, whose directions can be computed by taking the null space of the Jacobian matrix, **J** (i.e., an orthogonal set of eigenvectors, **e_i_**). For each of the 100 samples within one cycle, the individual mean-free finger forces over those cycles were projected onto these directions (UCM space), summed, and normalized by the 15% MVC_IMRL_ value and the number of degrees of freedom (DOF) to estimate the amount of variance per DOF in the UCM space (Equation 2):


(2) $${\text{V}}_{\text{UCM}}(\text{t})=\frac{\sum_{\text{j}=1}^{{\text{N}}_{\text{cycles}}}{\left|\sum_{\text{i}=1}^{\text{n}-\text{p}}\left(e_{\text{i}}\cdot m(t)\right)\cdot e_{\text{i}}\right|}^{2}}{(\text{n}-\text{p}){\text{N}}_{\text{cycles}}\cdot 0.15\cdot {\text{MVC}}_{\text{IMRL}}}$$

where ***n*** = 4 is the number of DOFs of individual finger forces, and ***p*** = 1 is the number of DOFs of the performance variable (F_TOT_). Analogously, the amount of variance per DOF orthogonal to the UCM (ORT space) was estimated (Equation 3):


(3) $${\text{V}}_{\text{ORT}}(\text{t})=\frac{\sum_{\text{j}=1}^{{\text{N}}_{\text{cycles}}}{\left|m(t)-\sum_{\text{i}=1}^{\text{n}-\text{p}}\left(e_{\text{i}}\cdot m(t)\right)\cdot e_{\text{i}}\right|}^{2}}{{\text{pN}}_{\text{cycles}}\cdot 0.15\cdot {\text{MVC}}_{\text{IMRL}}}$$

Then, the time-series of synergy index, ΔV(t), were calculated as the difference between V_UCM_ and V_ORT_ normalized by the total variance for each of the 100 samples as follows (Equation 4):


(4) $$\Delta \text{V}(\text{t})=\frac{{\text{V}}_{\text{UCM}}(\text{t})-{\text{V}}_{\text{ORT}}(\text{t})}{({\text{V}}_{\text{UCM}}\times 3+{\text{V}}_{\text{ORT}}\times 1)/4}$$

#### fNIRS Data Analysis

The raw fNIRS data, namely, the optical intensity (**I**), were filtered using discrete cosine transformation with a frequency ranging from 0.01 Hz to 0.5 Hz to eliminate instrumental and surrounding noises (Scholkmann et al., 2014; Shin et al., 2017). Prior to the variable computation, the signals from 48 channel qualities were estimated by computing the coefficient of variation (CV) of the optical intensity (CV_I_). Any channel that showed either high CV_I_ (i.e., CV_I_ > 40) or low optical intensity (**I** < 10) was rejected from further processing (Shin et al., 2017). ΔHbO was calculated using the modified Beer-Lambert law (Delpy et al., 1988). The averaged ΔHbO ($\bar{\text{ΔHbO}}$), which represents the changes in oxy-hemoglobin, O_2_Hb, concentration in each channel, was calculated by averaging ΔHbO signals across samples (i.e., duration by sampling frequency) in the selected phase for condition and participant, separately. Pearson’s correlation coefficients (*r*) between the *i*-th and *j*-th channels of ΔHbO signals were calculated using Equation 5 and then averaged across the three trials:


(5) $$\underline{r}=\text{ρ}({\text{ΔHbO}}_{\text{i}},{\text{ΔHbO}}_{\text{j}})=\frac{\sum_{\text{f=1}}^{\text{n}}({\text{ΔHbO}}_{\text{i,j}}-\bar{{\text{ΔHbO}}_{\text{i}}})({\text{ΔHbO}}_{\text{j,f}}-\bar{{\text{ΔHbO}}_{\text{j}}})}{\sqrt{\sum_{\text{f=1}}^{\text{n}}{({\text{ΔHbO}}_{\text{i,f}}-\bar{{\text{ΔHb}}_{\text{i}}})}^{2}}\sqrt{\sum_{\text{f=1}}^{\text{n}}{({\text{ΔHbO}}_{\text{j,f}}-\bar{{\text{ΔHbO}}_{\text{j}}})}^{2}}}$$

where ρ(·) denotes the Pearson’s correlation coefficient, ΔHBO_*i*_ and $\bar{{\text{ΔHbO}}_{\text{i}}}$ represent the ΔHbO signal at the *i*-th channel and the averaged ΔHbO of the corresponding channel, respectively. The total number of channels was 48; thus, the number of *channels* was 1,128 (i.e., combinations of 48 channels and a set of ΔHbO_*i*_ and ΔHbO_*j*_). The average magnitude of *r*(*r*) in each pair of channels was computed by separately averaging across participants for each frequency condition. The statistical significances of *r*(*p*) for all pairs were computed using one-sample *t*-test followed by log-transformation using Fisher’s *z*-transformation.

**Figure 2:**
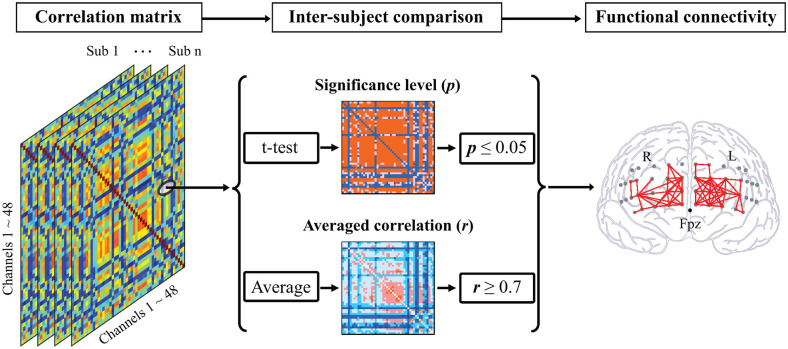
Illustration of functional connectivity analysis. A correlation matrix was calculated for each participant composed of Pearson’s correlation coefficients between each two channel pairs. For comparison within participants, the statistical significances of correlation for all pairs were computed based on a one-sample *t*-test followed by the log-transformation using Fisher’s *z*-transformation. By averaging the correlation matrices of all participants, the functional connectivity between two channels was identified as the high correlation coefficient (*r* ≥ 0.7) and the statistical significance (*p* ≤ 0.05).

One-sample *t*-test was performed to check the consistency of intra-channel correlations for all participants. The number of significant functional connections (FC_N_) was quantified based exclusively on the following criteria (Yu et al., 2020): (1) statistical significance at *p* ≤ 0.05 with the Bonferroni *p*-value correction method; and (2) high correlation coefficient, *r* ≥ 0.7 (Figure 2). In addition, the FC_N_ between channels located in the intra- and inter-hemispheres were counted separately.

### Statistics

A standard description of parametric statistics was used, and the data presented as means and standard errors. A parametric repeated-measures analysis of variance (ANOVA) with between-subject factors of *frequency* (three levels: 0.1, 1, and 2 Hz) and *phase* (two levels: F_DW_ and F_UP_) was performed on the RP, (CV_peak_ and CV_trough_), averaged ΔHbO at each of 48 channels, and FC_N_ (Hypotheses 1 and 3). All factors were selected for the particular statistical tests. Mauchly’s sphericity test was used to confirm or reject the assumptions of sphericity. Greenhouse-Geisser corrections were used when the sphericity assumption was rejected. The statistical power for all comparisons from the pool of nine participants was computed, and in all cases, the power was >0.7. For the pair-wise comparison, the changes in the magnitude of the estimated variables were presented using post calculation of the effect size (partial eta-squared, $\eta_{\text{p}}^{2}$) and the lower and upper 95% confidence intervals (CIs). In addition, the normality of the measured data was assessed by the coefficients of curvature (skewness) and elongation (kurtosis) upon the statistical significance. The level of significance for all statistical tests was set at *p* < 0.05.

To test the influence of *frequency* on the time-series variables from the UCM computation, including V_UCM_(t), V_ORT_(t), and ΔV(t), one-dimensional statistical parametric maps (SPM) with repeated-measured ANOVAs (SPM{F}, Pataky et al., 2016) were performed for the F_UP_ and F_DW_ phases separately (hypothesis 2). SPM analyses were conducted using open-source software^1^ (Pataky, 2012). A critical threshold for the SPM analyses was computed based on the random field theory (Worsley et al., 2004), which was set at *α* = 0.05. When the SPM curve crossed the critical threshold, the difference was deemed to be a significant effect of *frequency* at particular time points of the curves. In case of significant effects of *frequency*, follow-up SPM{t} paired *t*-tests were performed. In particular, linear regression models were applied to each sample of the normalized data set (i.e., 100 samples) to generate the SPM{F} curve across three frequency conditions and the SPM{t} curve for the pairs of two frequency conditions.

## Results

### Accuracy of Cyclic Force Production

The group means and standard deviations of the RP determined using the pooled data over F_UP_ and F_DW_ phases were 2.68° ± 0.10°, 2.91° ± 0.16°, and 3.16° ± 0.20° under the conditions of 0.1 Hz, 1 Hz, and 2 Hz, respectively. These results imply that the two sets of cyclic force data were close to in-phase synchronization for all three conditions. Two-way repeated-measured ANOVAs performed with the factors of *frequency* (three levels: 0.1, 1, and 2 Hz) and *phase* (two levels: F_DW_ and F_UP_) showed no significant main effects and factor interaction effects on the RP. The average CV values across subjects were negative for all three frequency conditions, representing force undershoots in most cases. In particular, the amount of force undershoots increased with frequency, especially for the CV_peak_. These observations were supported by the Bayesian one-way repeated-measures ANOVAs, separately on CV_peak_ and CV_trough_, with *frequency* as a factor, which confirmed a significant effect of this factor on CV_peak_ (*F*_(1,8)_ = 27.19, *p* < 0.01, $\eta_{\text{p}}^{2}$ = 0.90), but not on CV_trough_. *Post hoc* pairwise comparison further confirmed that the average CV_peak_ was −0.17 at 0.1 Hz [CI: (−0.23 −0.10), *p* < 0.05], > −0.42 at 1 Hz [CI: (−0.56 −0.29), *p* < 0.05], and −0.58 at 2 Hz [CI: (−0.73 −0.43), *p* < 0.05]. Lastly, the coefficients of skewness and kurtosis were −0.79 and 1.14, respectively.

### Multi-finger Coordination Indices

**Figure 3:**
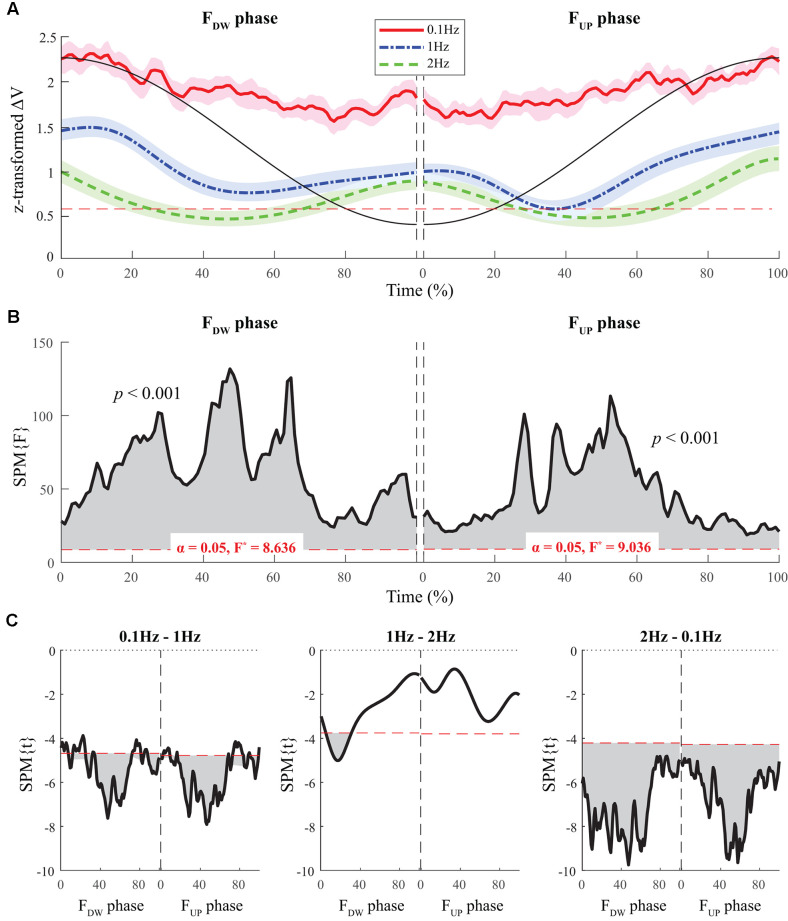
The sample data of total force (black solid line) and the time profiles of averaged ΔVs across participants for three frequency conditions are presented with standard deviations shades during the F_DW_ (*left panel*) and F_UP_ (*right panel*) phases. Red solid-, blue dashed-single dotted-, and green dashed lines present ΔV time-profiles for 0.1, 1, and 2 Hz, respectively. The horizontal dotted line in red color indicates a critical value of *z*-transformed ΔV (i.e., per-dimensional V_UCM_ equals V_ORT_), which determines the existence of the synergy **(A)**. Statistical parametric mapping SPM{F} trajectories on ΔVs (black solid line) with repeated measures analysis of variance (ANOVA) are presented for the F_DW_ (*left panel*) and F_UP_ (*right panel*) phases. The horizontal dotted line in red color indicates the threshold of critical random field theory at α = 0.05 **(B)**. *Post hoc* pairwise *t*-tests (SPM{t}) on ΔVs are presented. The horizontal dotted lines in red color indicate the threshold of critical random field theory at *p* < 0.05 **(C)**.

The average time profiles of ΔV with standard deviations across subjects for the F_UP_ and F_DW_ phases are presented in Figure 3. For both F_UP_ and F_DW_ phases, a U-shape profile of ΔVs over time was observed under the 1 Hz and 2 Hz conditions, while ΔV was consistently higher under the 0.1 Hz condition than under the other two conditions (Figure 3A). SPM analysis with repeated-measures ANOVA performed separately on the F_UP_ and F_DW_ phases showed significant effects of *frequency* on ΔV over the entire time period (Figure 3B). In particular, the significant effect of *frequency* was prominent in the middle of the time period for both the F_UP_ and F_DW_ phases. *Post hoc* pairwise SPM *t*-tests confirmed ΔV at 0.1 Hz was greater than that at 1 Hz and 2 Hz for both F_UP_ and F_DW_ conditions, especially when SPM{F}crossed the critical value (Figure 3C).

**Figure 4:**
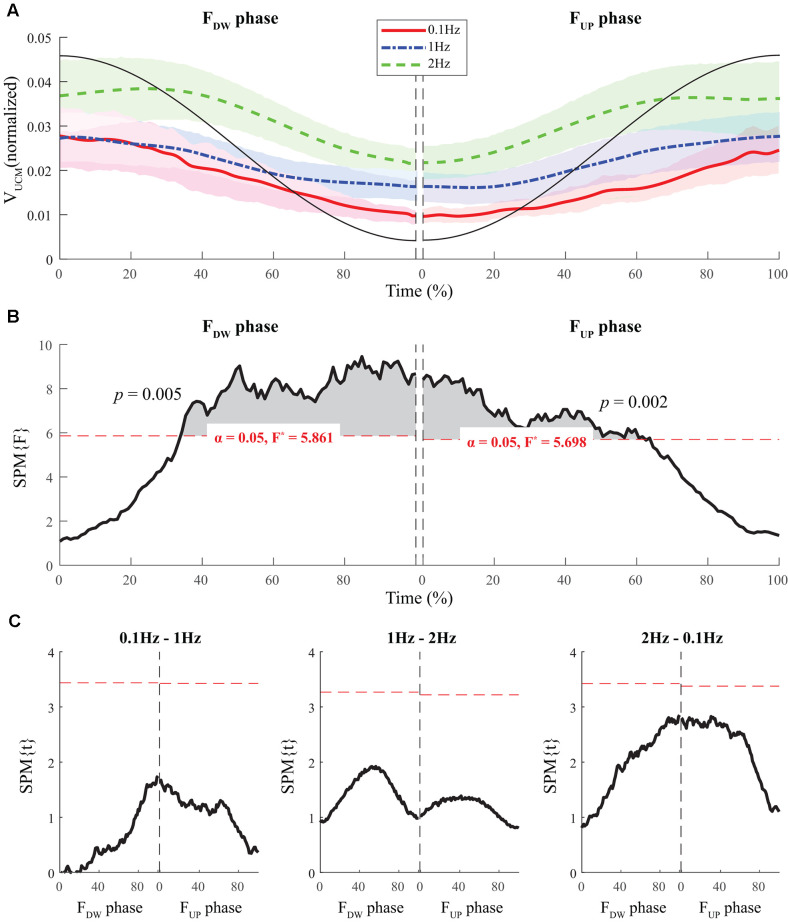
The sample data of total force (black solid line) and the time profiles of the mean values of normalized V_UCM_ across participants for three frequency conditions are presented with standard deviations shades during the F_DW_ (*left panel*) and F_UP_ (*right panel*) phases such that red solid-, blue dashed-, and green dashed-lines present ΔV time-profiles for 0.1, 1, and 2 Hz, respectively **(A)**. SPM{F} trajectories on V_UCM_ (black solid line) with repeated measures ANOVA are presented for the F_DW_ (*left panel*) and F_UP_ (*right panel*) phases. The horizontal dotted line in red color indicates the threshold of critical random field theory at α = 0.05 **(B)**. *Post hoc* pairwise *t*-tests (SPM{t}) on V_UCM_ are presented. The horizontal dotted lines in red color indicate the threshold of critical random field theory at *p* < 0.05 **(C)**.

**Figure 5:**
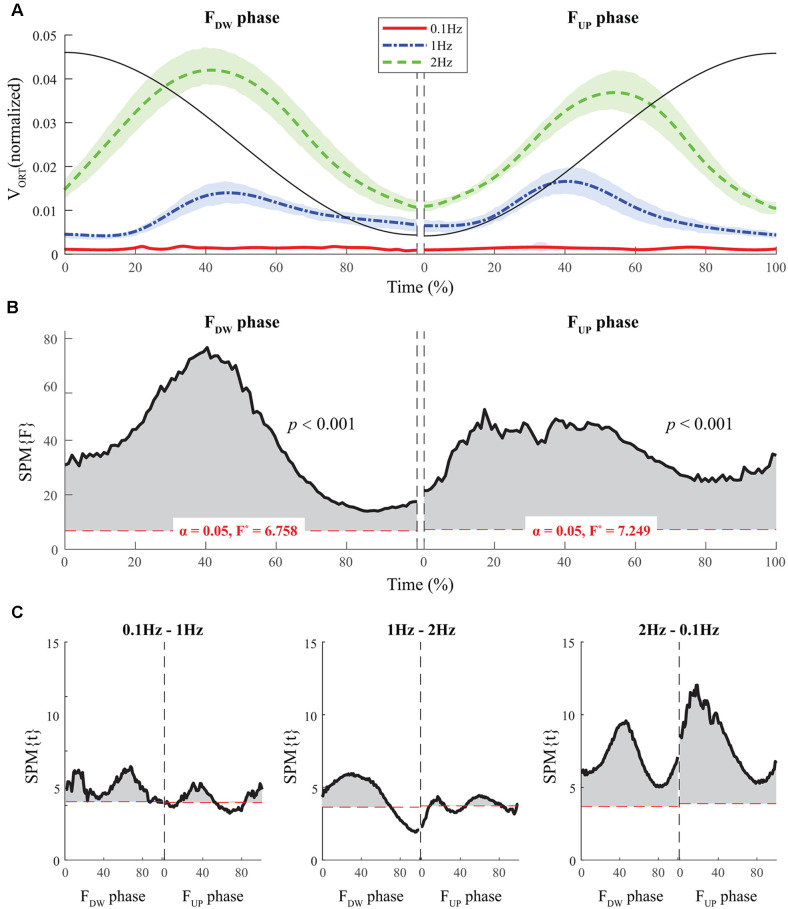
The sample data of total force (black solid line) and the time profiles of the mean values of normalized V_ORT_ across participants for three frequency conditions are presented with standard deviations shades during the F_DW_ (*left panel*) and F_UP_ (*right panel*) phases such that red solid-, blue dashed-, and green dashed-lines present ΔV time-profiles for 0.1, 1, and 2 Hz, respectively **(A)**. SPM{F} trajectories on V_ORT_ (black solid line) with repeated measures ANOVA are presented for the F_DW_ (*left panel*) and F_UP_ (*right panel*) phases. The horizontal dotted line in red color indicates the threshold of critical random field theory at α = 0.05 **(B)**. *Post hoc* pairwise *t*-tests (SPM{t}) on V_ORT_ are presented. The horizontal dotted lines in red color indicate the threshold of critical random field theory at *p* < 0.05 **(C)**.

Normalized time functions (i.e., 100-time points) of the two variance components, V_UCM_(t) and V_ORT_(t), were computed. The time profiles between the two variances were different, such that V_UCM_(t) and V_ORT_(t) were related to the magnitude of the total force (|F_TOT_|) and the absolute value of the force derivative (|dF_TOT_/dt|, i.e., inverted U-shape), respectively. To facilitate comparisons between the three frequency conditions, the condition with a larger frequency showed higher V_ORT_ values for both F_UP_ and F_DW_ phases (Figure 5A). However, little difference in V_UCM_ magnitude was observed between frequency conditions (Figure 4A).

The SPM analysis with repeated-measures ANOVA showed significant effects of *frequency* on both V_UCM_ (Figures 4B,C) and V_ORT_ (Figures 5B,C), with a stronger effect on the latter than on the former. In particular, a strong effect of *frequency* on V_ORT_ was observed in the middle portion of the period for both F_UP_ and F_DW_ phases, where the peak values of the force derivative (dF/dt) were observed (Figure 5B). For the SPM{F} for V_UCM_, a relatively strong *frequency* effect was observed when the force magnitude was small, such as in the latter portion of the F_DW_ and the early portion of the F_UP_ phases. *Post hoc* pairwise SPM *t*-tests confirmed that V_ORT_ trend for both F_UP_ and F_DW_ conditions was as follows: 0.1 Hz > 1 Hz 2 Hz (Figure 5C).

### Oxygenation Indices

**Figure 6:**
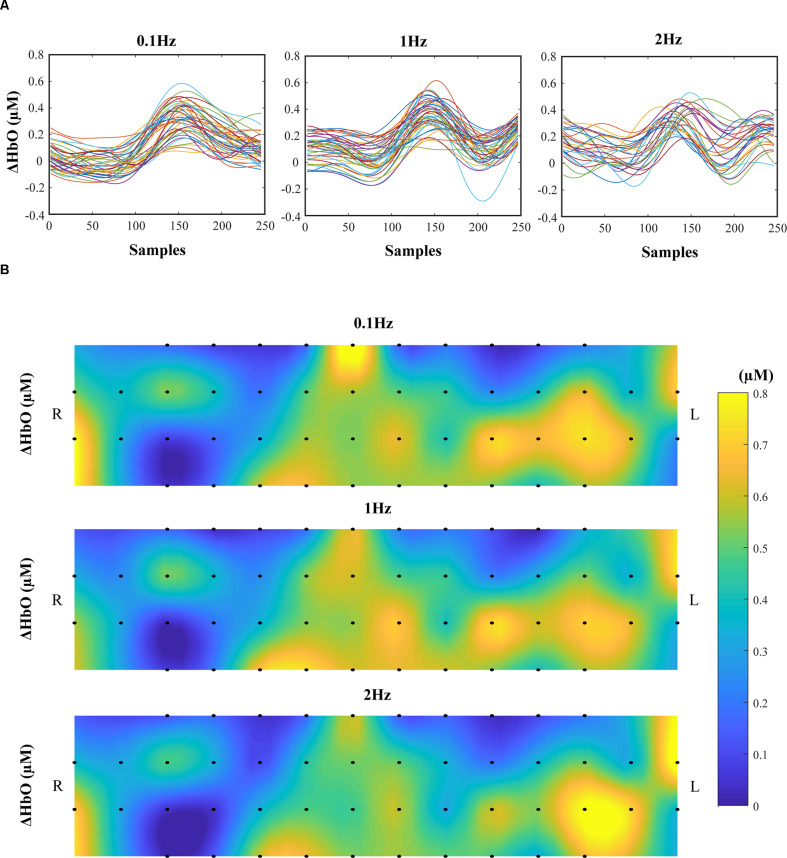
The concentration changes in oxy-hemoglobin (ΔHbO). The time profiles of ΔHbO of all 48 channels are presented in 0.1 Hz (*left panel*), 1 Hz (*middle panel*), and 2 Hz (*right panel*). The data are from a representative participant **(A)**. Average ΔHbOs across time samples and participants for 48 channels were presented. The *top panels* show the average ΔHbO for 0.1 Hz, the *middle panels* for 1 Hz, and the *bottom panels* for 2 Hz condition. The color bar encodes the strength of ΔHbO, and the bar length corresponds to the ΔHbO values between 0 μM and 0.8 μM **(B)**.

**Figure 7:**
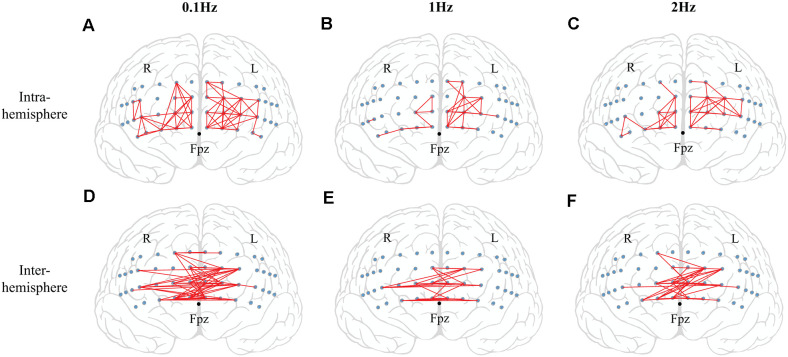
The results of functional connectivity (FC_N_). The significant functional connectivity (FC_N_) between two channel pairs is shown by red lines. The small blue circles indicate the locations of fNIRS channels. The *top panel* shows the significant functional connectivity in the intra-hemisphere for 0.1 Hz (*left panel*) **(A)**, 1 Hz (*middle panel*) **(B)**, and 2 Hz (*right panel*) **(C)** conditions. The *bottom panel* indicates the significant functional connectivity in the inter-hemisphere for 0.1 Hz (*left panel*) **(D)**, 1 Hz (*middle panel*) **(E)**, and 2 Hz (*right panel*) **(F)** conditions.

There were no significant effects on *frequency* and *phase* on ΔHbO for all 48 channels; on average, ΔHbO was around 0.3 μM (Figure 6A). Higher ΔHbO values were observed in the left hemisphere than in the right hemisphere, and the middle portion of the left hemisphere showed stronger ΔHbO for all three frequency conditions (Figure 6B). In addition, the FC_N_ under each frequency condition was determined by one-sample *t*-tests with the criteria of *p* ≤ 0.05 and *r* ≥ 0.7. Note that a prior analysis showed no significant difference between FC_N_ for F_UP_ and F_DW_ phases; thus, the data presented in Figure 7 and Table 1 were quantified using pooled data across F_UP_ and F_DW_ phases for each frequency condition. The number of significant pairs (i.e., FC_N_) decreased with an increase in frequency (128, 50, and 61 under the 0.1 Hz, 1 Hz, and 2 Hz conditions, respectively; Table 1). Similarly, both intra- and inter-hemispheric FC_N_ decreased with increasing frequency, where the intra-hemisphere FC_N_ was larger than the inter-hemisphere FC_N_ (Table 1).

**Table 1:** The number of significant functional connectivity (FC_N_).

Frequency condition		0.1 Hz	1 Hz	2 Hz
Intra-hemisphere	Left	42	19	24
	Right	33	8	11
Inter-hemisphere		53	23	26
Total		128	50	61

## Discussion

In this study, we investigated the frequency-dependent changes in the synergistic actions of four fingers and a set of indices for prefrontal cortex oxygenation during isometric cyclic-force-production tasks. Both expected and unexpected outcomes were also observed. First, the accuracy of the performance decreased with an increase in the frequency (hypothesis 1). In particular, a less accurate performance in the high-frequency condition was not a consequence of the change in the relative phase, but it was mainly caused by larger deviations of peak or trough forces across cycles. Second, the synergy indices for the total force stabilization were larger in the low-frequency condition (0.1 Hz) than in the other two higher frequency conditions, which was dominantly affected by the increased V_ORT_ in the higher frequency conditions (hypothesis 2). Contrary to our expectation, the concentration of O_2_Hb remained unchanged under all frequency conditions, while the functional connectivity decreased with an increase in the frequency of cyclic force production, which partially supported hypothesis 3.

### Frequency-Dependency of Stability in Cyclic Force Production

The main premise behind the concept of *synergy* is a crucial feature of all intentional movements performed by redundant systems that possibly represent the system stability (Latash, 2008a). Specifically, stable performance is coined to address the flexible patterns while compensating for errors among a redundant or, to be specific, abundant set of elements considering the mechanics of a particular task, whereby the solution family is organized in such a way that the actions of elements are varied to maintain the performance variables in an unchanged (or stabilized) state (Reschechtko et al., 2014; Ambike et al., 2016). One of the straightforward motivations of the current study is the curiosity as to whether the strategy and consequences of the organization of the solution family (i.e., combinations of four finger forces) change with different frequencies during cyclic force production. The current results of the inconsistent peak or trough force production (i.e., higher CV) were accompanied by a small synergy index at relatively high-frequency conditions, which is in line with the previous findings of the frequency dependence of various motor behaviors from the perspective of variability in muscle activation (Lewis et al., 2001; Huang et al., 2021), sensory information processing (Almeida et al., 2002), etc.

From a computational point of view, the synergy index refers to the relative magnitudes of V_UCM_ and V_ORT_ with respect to the total variance (V_TOT_). In other words, less flexible combinations of elements (V_UCM_) could be associated with a larger synergy index if the error variance (V_ORT_) is significantly small. The current results showed that a strong statistical effect of *frequency* conditions was mainly observed in V_ORT_, while the statistical effect on V_UCM_ was relatively weak, resulting in small synergy indices under high-frequency conditions. These results reflect the fact that the elements (i.e., force modes, **m**) had a dominant positive covariance at higher frequencies. The dominant positive co-variation between elements possibly causes inconsistent peak and trough force values, whereby performance errors increase accordingly. On the contrary, a strong negative co-variation between elements would serve as an error compensation strategy as to the total force stabilization, which was dominantly shown under the small frequency condition (0.1 Hz; Tseng et al., 2006).

However, we hesitate to strongly claim that the low values of synergy indices under high-frequency conditions represent weakened force stabilization. In Figure 5A, it was clearly observed that the time patterns of the orthogonal complement of variance (V_ORT_) fit well with the rate of force changes (dF/dt, not shown in Figure), and this phenomenon is well described by the mathematical model, that is, the linear model proposed by Gutman (Gutman and Hagander, 1985; Latash et al., 2002b) and other experimental outcomes reported previously (Friedman et al., 2009). Because the direction of force changes results only from the combinations of elements within the subspace of the orthogonal complement to the null space (i.e., UCM space), the variance within the orthogonal subspace strongly depends on the scaling of the rate of force change. Thus, it is plausible that the increment of frequency naturally requires changes in the V_ORT_, which results in less accurate (or more variable) force values at the peak and trough of the force profile. Contrary to our expectations, the results of the RP were close to the in-phase synchronization under all frequency conditions, which suggests that the phase difference between the produced and constrained force values was not the primary source of error variance. Of course, a less performance accuracy in the high-frequency condition is also caused by the inertial or biomechanical factors such as the bandwidth of muscle activation (Van Boxtel et al., 1998), electromechanical delay (Cavanagh and Komi, 1979), etc. In particular, the consideration of the inertial properties of body segments would be critical to avoid spurious interpretation of behavioral changes with frequency in dynamics. However, the relative contributions of the central and peripheral components to the performance accuracy remain unknown, even with the current results; thus, future research will have to ascertain the relationship between the inertial/biomechanical properties of the human body and neural response with various movement frequencies. Nevertheless, we have to find an alternative approach to answer the following question: *What are the neural activities that cause changes in synergy indices for multi-finger force production with different frequencies of cyclic force production?*

### Frequency-Dependency of Prefrontal Oxygenation and Its Relationship With Synergy

In the current study, we measured the hemodynamic responses in the prefrontal cortex while acquiring individual finger forces during cyclic force production. We quantified two indices of prefrontal hemodynamics, including ΔHbO and functional connectivity. Note that ΔHbO is indicative of the amount of localized oxygen consumption, and the functional connectivity represents significant connections (i.e., concurrent changes in oxygen consumption) between anatomically separated brain regions within the prefrontal structure. The current results showed non-parallel changes in the indices with the frequencies of cyclic force production, that is, O_2_Hb concentration remained unchanged, whereas the functional connectivity (i.e., the number of significant connections) decreased with frequency. In particular, it might seem counterintuitive that the ΔHbO was not frequency-dependent, and these results are in stark contrast to previous findings regarding the frequency-dependency of cortical activations (i.e., the higher the frequency, the larger the cortical activation; Jenkins et al., 1997; Turner et al., 1998; Lutz et al., 2004), which presumably increased the oxygen concentration (Scholkmann et al., 2014). Oxygen is definitely a source of energy or cost for brain activities (McKenna et al., 2012). This difference between the results of the current and previous studies may have been caused by dissimilar experimental tasks and the focused region within the brain. Nevertheless, the unchanged index of oxygen consumption, particularly in the prefrontal cortex, is unlikely the “cost” for the current experimental tasks. On the contrary, concurrent prefrontal activation seems to be the frequency-dependent index for the current motor tasks, consistent with previous findings (Cordes et al., 2001; Salvador et al., 2008).

The current motor tasks required the subjects to use visual feedback. In other words, the values of cyclic force trajectories were prescribed through the template on the screen, and the subjects were asked to produce a total finger force such that the produced force with a cursor on the screen chased a prescribed target trajectory. For the low-frequency condition (e.g., 0.1 Hz condition), the subjects had enough time to utilize the feedback of visual or proprioceptive information, whereby it is possible that feedback-based force production is a critical factor in achieving a better performance accuracy under low-frequency than under high-frequency condition. The feedback-based error compensation strategy between finger forces may be less (or not) used during high-frequency conditions because of the limited time to use and process sensory feedback signals, which may result in a relatively large deviation and variance (i.e., error variance, V_ORT_). Further, this speculation is supported by the dominant positive co-variation of finger forces (Goodman et al., 2005) under high-frequency conditions. By combining the results of hemodynamic responses and patterns of finger force co-variation, the error variance in a particular task could be linked to the functional connectivity of the prefrontal cortex, and not the magnitude of oxygen saturation. Indeed, it has been reported that the metabolism of brain activation with other brain imaging techniques (e.g., positron emission tomography, magnetic resonance imaging, etc.) showed a non-linear relationship with the degree of connectivity (Neubauer and Fink, 2009; Tomasi et al., 2014; Tomasi and Volkow, 2019) and that concurrent brain activation is caused by a combined effect of anatomical connectivity and synaptic efficiency (Buckner, 2010). Furthermore, unchanged oxygen saturation in the prefrontal cortex was observed during continuous movements with different properties (Jenkins et al., 1997; Kim et al., 2005), which is in line with the current results. However, the methodologies for the acquisition of brain activities in the current and previous studies are different, and functional connectivity is assumed to be an indirect quantification of brain activation. A possible interpretation, however, of the increase in functional connectivity, combined with unchanged oxygen consumption and small error variance for the stabilization of total finger forces in the low-frequency condition, is that the behavioral stability could be associated with the efficient organization of prefrontal activation.

This interpretation is in line with theories and computational models used for controlling a multi-element system. The computational model proposed by Todorov and Jordan (2002) (i.e., stochastic optimal control model) claims that the controller’s effort is not dedicated to the deviation of elements that do not interfere with critical mechanics but is more concerned with error correction than with the required mechanics. In other words, the energy/cost function may be associated with minimizing performance error rather than organizing variable combinations of solutions. This idea is conceptually similar to the UCM hypothesis based on the principle of motor abundance, which describes a crucial feature of the co-variation patterns between elements (Todorov and Jordan, 2003; Martin et al., 2009). In turn, the experimental outcomes based on the UCM hypothesis and optimal control model provide evidence supporting the current claim, both of which consistently suggest the minimization of a fraction of error variance as the controller’s strategies to govern the multi-element system. In this regard, the current findings show that the changes in the concurrent prefrontal cortex activation (i.e., functional connectivity) are affected by the frequency of cyclic actions, which is associated with the modulation of error variance (V_ORT_), but not with the variance corresponding to the required mechanics (V_UCM_), that is, the total finger forces.

To the best of our knowledge, the current study is the first work to uncover the effect of frequency on the multi-finger synergies in relation to the hemodynamic response in the prefrontal cortex, which is interconnected with diverse brain areas, including the cortical, subcortical, and brainstem regions (Miller and Cummings, 2017). In particular, the experimental findings in this study would contribute to the current knowledge on the neural origin of finger-force combinations for the stabilization of salient performance variables. However, this study has several limitations. First, the measurement of hemodynamics using fNIRS yields is more beneficial in terms of portability, cost-efficiency, and absence of hazardous effects of radiation. On the other hand, quantifying hemodynamic indices through fNIRS provides an indirect measurement of neuronal activity. Second, fNIRS may not be suitable for the direct monitoring of subcortical activity, even though the motor cortico-basal ganglia circuit originates from the prefrontal cortex (Fuster, 2015). As the formation of synergy for stable performance is assumed to be a function of subcortical structures (i.e., trans-thalamic loop; Rispal-Padel et al., 1981; Park et al., 2012b; Latash and Huang, 2015), future studies will have to ascertain the validity of fNIRS measures by comparing them with those of other brain imaging data during behavioral tasks. Also, the apparent drawback of the participant recruitment was that the gender of all participants was male with a small sample size. Although the results of previous studies claimed that the stability indices are not a gender-dependent (i.e., strength-dependent) quantity if the participants have no medical history of the peripheral and neurological disorder (Zhang et al., 2007; Friedman et al., 2009), the extremely swayed recruitment as to the gender may cause uncertainty and hamper the interpretation and generalization of the current messages. Lastly, given the diverse factors related to the coordinative action in humans, future studies will have to investigate whether the current message would be valid with age-related changes, gender, and other biological indices.

## Data Availability Statement

The datasets measured during the current experiments are available from the corresponding author (JP) on reasonable request.

## Ethics Statement

The studies involving human participants were reviewed and approved by The Seoul National University Institutional Review Board (IRB). The patients/participants provided their written informed consent to participate in this study.

## Author Contributions

All of the authors contributed to the design of the study, interpretation of the data, and writing of the manuscript. DX performed the experiment, analyzed data, and participated in the writing of the first draft. NS and SL took a significant share of the experiment and data analysis. JP conceptualized the experimental and computational methods, wrote the first draft, and edited and reviewed the manuscript. All authors contributed to the article and approved the submitted version.

## Conflict of Interest

The authors declare that the research was conducted in the absence of any commercial or financial relationships that could be construed as a potential conflict of interest.

## Publisher’s Note

All claims expressed in this article are solely those of the authors and do not necessarily represent those of their affiliated organizations, or those of the publisher, the editors and the reviewers. Any product that may be evaluated in this article, or claim that may be made by its manufacturer, is not guaranteed or endorsed by the publisher.

## Footnotes

^1^ www.spm1d.org
